# MicroRNA-31 functions as a tumor suppressor by regulating cell cycle and epithelial-mesenchymal transition regulatory proteins in liver cancer

**DOI:** 10.18632/oncotarget.3512

**Published:** 2015-03-10

**Authors:** Hyung Seok Kim, Kyo Sun Lee, Hyun Jin Bae, Jung Woo Eun, Qingyu Shen, Se Jin Park, Woo Chan Shin, Hee Doo Yang, Mijung Park, Won Sang Park, Yong-Koo Kang, Suk Woo Nam

**Affiliations:** ^1^ Lab of Oncogenomics, Department of Pathology, College of Medicine, The Catholic University of Korea, Seoul, Republic of Korea; ^2^ Functional RNomics Research Center, The Catholic University of Korea, Seoul, Republic of Korea; ^3^ Department of Orthopedic Surgery, College of Medicine, The Catholic University of Korea, Gyeonggi-do, Korea; ^4^ Cancer Evolution Research Center, Catholic University of Korea, Seoul, Republic of Korea

**Keywords:** Hepatocellular carcinoma, microRNA-31, CDK2, HDAC2, cell cycle

## Abstract

MicroRNA-31 (miR-31) is among the most frequently altered microRNAs in human cancers and altered expression of miR-31 has been detected in a large variety of tumor types, but the functional role of miR-31 still hold both tumor suppressive and oncogenic roles in different tumor types. MiR-31 expression was down-regulated in a large cohort of hepatocellular carcinoma (HCC) patients, and low expression of miR-31 was significantly associated with poor prognosis of HCC patients. Ectopic expression of miR-31 mimics suppressed HCC cell growth by transcriptional deregulation of cell cycle proteins. Additional study evidenced miR-31 directly to suppress *HDAC2* and *CDK2* expression by inhibiting mRNA translation in HCC cells. We also found that ectopic expression of miR-31 mimics reduced metastatic potential of HCC cells by selectively regulating epithelial-mesenchymal transition (EMT) regulatory proteins such as N-cadherin, E-cadherin, vimentin and fibronectin. HCC tissues derived from chemical-induced rat liver cancer models validated that miR-31 expression is significantly down-regulated, and that those cell cycle- and EMT-regulatory proteins are deregulated in rat liver cancer. Overall, we suggest that miR-31 functions as a tumor suppressor by selectively regulating cell cycle and EMT regulatory proteins in human hepatocarcinogenesis providing a novel target for the molecular treatment of liver malignancies.

## INTRODUCTION

Hepatocellular carcinoma (HCC) is one of the most deadly cancers worldwide and has no effective treatment [1]. The major risk factors causing liver cancer are variable including infection of HBV or HCV, alcoholic liver disease, and non-alcoholic fatty liver disease, and these risk factors lead to development of cirrhosis in the most of liver cancer patients. Even though the overall survival rate of liver cancer patients was increased thanks to recent studies, there are still no systemic treatment showing consistently efficient for liver cancer [2]. Accumulating evidences have suggested that the loss of several tumor suppressors and aberrant regulation of cellular growth signaling, such as the ERK/MAPK pathway and Wnt/beta-catenin pathway, are associated with liver tumorigenesis, but the molecular pathogenesis of HCC remains poorly understood [3]. In recent years, aberrant regulation of non-coding RNAs (ncRNAs) has been proposed to be associated with hepatocarcinogenesis. The ncRNAs can be divided into two major categories, long non-coding RNAs and short non-coding RNAs [4]. To date, the most extensively studied small RNAs in cancer are microRNAs (miRNAs). Elegant studies over the past 15 years have defined an intricate mechanistic basis for miRNA-mediated silencing of target gene expression through the RNA-induced silencing complex, which employs Argonaute family proteins to cleave target mRNA transcripts or inhibit the translation of that mRNA [4].

MiRNAs are short non-coding RNAs with 21 ~ 25 nucleotides in length which target mRNAs for degradation or translational repression by direct binding to the 3′-untraslated region (3′-UTR) of mRNAs [5]. The involvement of miRNAs in cancer pathogenesis is well established, as miRNAs can behave as oncogenes or tumor suppressor genes depending on the cellular function of their targets. Moreover, activation or suppression of specific miRNA families are mechanisms through which oncogenes, such as Myc, or tumor suppressor genes, such as p53, induce or inhibit tumorigenesis. Although their functions are still elusive, it is obvious that miRNAs play an important role in initiation of cancer and progression by regulation of tumor suppressor genes or oncogenes [6]. In cancer studies, numerous reports have linked deregulation of miRNA expression to liver cancers. Down-regulations of miRNAs are observed in liver cancer and the target genes regulated by these miRNAs are possibly acting as oncogenes. On the contrary, over-expressed miRNAs may have oncogenic function by targeting tumor suppressors in HCC. For instance, some miRNAs, such as miR-29, miR-21 and miR-221, has been reported to regulate tumor cell growth, apoptosis, migration and invasion by targeting proteins involved in those cellular pathways [7-9]. However, the roles of miRNA in hepatic carcinogenesis are complex; different studies have reported unique profiles, with only a few miRNAs in common, indicating the heterogeneity of HCC.

The expression of miR-31 varies from one cancer to another and thus its functional role is very diverse in different malignancies. Hence, the functional role of miR-31 is extremely complex and miR-31 can hold both tumor suppressive and oncogenic roles in different tumor types. The phenotype caused by aberrant miR-31 expression seems to be strongly dependent on the endogenous expression levels. For instance, miR-31 was significantly down-regulated in breast cancer and bladder cancer, thus expression of miR-31 was inversely correlated with metastasis and aggressiveness [10, 11]. In some cases, such as colon cancer and oral cancer, miR-31 was highly over-expressed [12, 13]. Furthermore, even though expression of miR-31 and its functions were extensively studied and well defined in many cancers, the role of miR-31 in human liver cancer is still unidentified. In this study, we showed that miR-31 expression was significantly down-regulated in a subset of HCCs, and the low expression of miR-31 was associated with a poor prognosis in HCC patients. Ectopic expression of miR-31 potentially suppressed cell growth via transcriptional inactivation of *HDAC2* and *CDK2*. In addition, overexpression of miR-31 mimics significantly abolished metastatic potential of HCC cells. Here we report that miR-31 functions as a tumor suppressor through the regulation of cell cycle and epithelial-mesenchymal transition (EMT) proteins in hepatocarcinogenesis.

## RESULTS

### MiR-31 is aberrantly down-regulated in HCC and its expression is associated with the poor prognosis of patients with HCC

**Figure 1:**
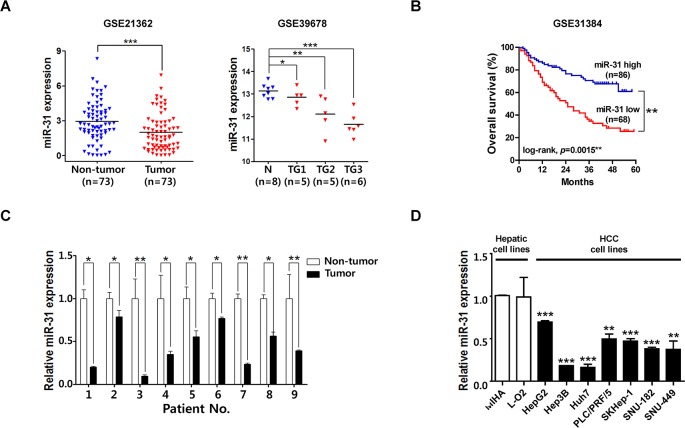
MiR-31 is down-regulated in hepatocellular carcinoma (A) Recapitulated miRNA expression levels of the large cohort of HCC patients. The miRNA microarray data were obtained from NCBI, GEO database (Accession No: GSE21362 and GSE39678). The miRNA expression of HCC patients were illustrated by scatter plots. The median expression is indicated by horizontal line. The microRNA expression levels are shown on the *y* axis (log2 intensity, *P<0.05; **P<0.005; ***P<0.001, Student's *t* test) (TG1; Edmonson grade I, TG2; Edmonson grade II, TG3; Edmonson grade III) (B) Kaplan-Meier survival curve of the GSE31384 dataset. The five year survival rate was significantly decreased in patient with low level of miR-31 expression in the tumor tissues (Log-rank *P* = 0.0015*) (C) The qRT-PCR analysis for 9 paired HCC tissues. MiR-31 was significantly down-regulated compared to corresponding non-tumor tissue. The expression of miR-31 was normalized to U6 snRNA (*P<0.05; **P<0.005, Student's *t* test) (D) The qRT-PCR analysis of miR-31 for hepatocellular carcinoma cell lines (n=7) and liver normal cell lines (n=2) (**P<0.005; ***P<0.001, Student's *t* test).

MiR-31 is among the most frequently altered miRNAs in human cancers and altered expression of miR-31 has been detected in a large variety of tumor types. For example, miR-31 down-regulation has been detected in several other malignancies, such as bladder, esophageal, ovarian, and prostate cancer as well as in glioma, leukemia, melanoma, and mesothelioma. However, increased expression of miR-31 has also been detected for example in colorectal, lung and pancreatic cancer, head and neck squamous cell carcinoma, and osteosarcoma [14]. For liver cancer, the only one study reported that miR-31 was over-expressed, but no correlation with clinicopathlogical features was found [15]. Thus, functional role of miR-31 in liver cancer is elusive and to be uncovered. Therefore, to validate the expression of miR-31 in liver cancer, we observed miR-31 expression in the large cohorts of HCC patients available from the National Center for Biotechnology Information (NCBI) and Gene Expression Omnibus (GEO) database (accession numbers GSE21362 and GSE39678), and the data were presented as scatter plots. Unlikely with previous observation in liver cancer study, miR-31 expression was significantly down-regulated in these two different HCC cohorts (Fig. 1A). Interestingly, in a subset of HCCs defined by Edmondson grade I (TG1, n = 5), grade II (TG2, n = 5), grade III (TG3, n = 6), miR-31 was gradually down-regulated in the progression of liver cancer (Fig. 1A, GSE39678). In addition, Kaplan-Meier survival curves of patients with HCC indicated that the 5-year overall survival (OS) rates of HCC patients with low miR-31 expression was significantly lower than that of HCC patients with high miR-31 expression (Fig. 1B). Next, to verify the suppression of miR-31 in HCC patients, miR-31 expressions of 9 randomly selected HCC tissues paired with adjacent non-cancerous liver tissues were investigated by quantitative real-time PCR (qRT-PCR). From this, all 9 HCC tissues exhibited significantly down-regulation of miR-31 in HCC (Fig.1C). Additionally, endogenous expression of miR-31 was investigated by qRT-PCR in nine different liver cell lines, including immortalized normal hepatic cell lines (Fig. 1D). The human liver cancer cell lines (Hep3B, Huh7, PLC/PRF/5, SK-Hep-1, SNU-182 and SNU-449) exhibited relatively low miR-31 expression levels compared to that of non-cancer cell lines (MIHA and L-O2). These results suggest that the expression of miR-31 is suppressed in HCC and its low expression associates with biological process of tumorigenesis and poor prognostic signs of HCC patients.

### Ectopic expression of miR-31 elicits a tumor-suppressor effect by regulating cell-cycle proteins in liver cancer cells

**Figure 2:**
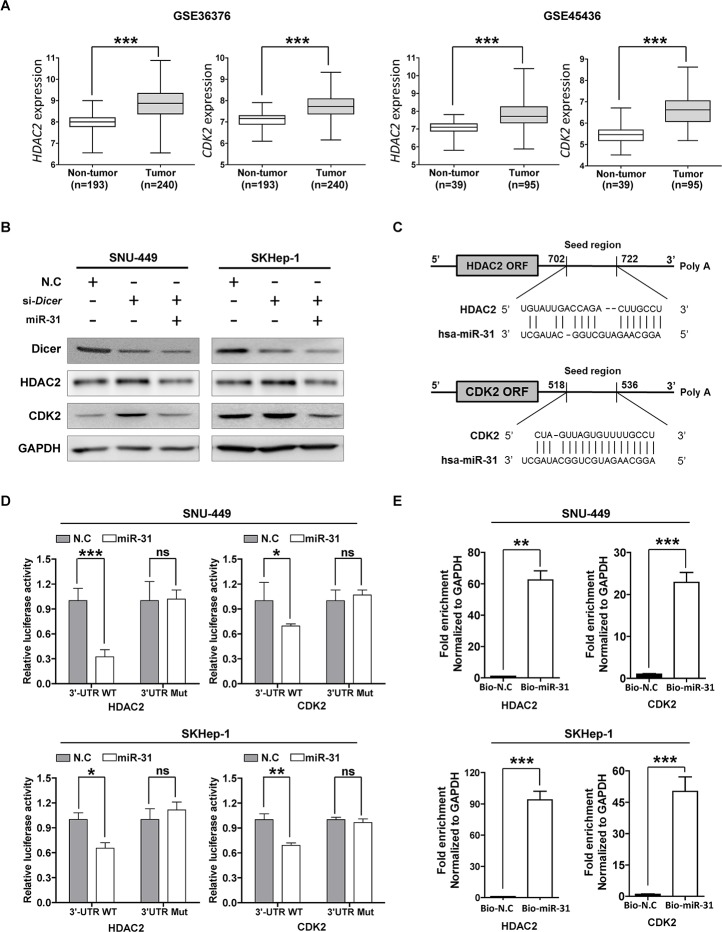
MiR-31 regulates HDAC2 and CDK2 expression by binding 3′-UTR in hepatocellular carcinoma (A) Differential expression of *HDAC2* and *CDK2* mRNAs from the mRNA microarray data obtained from NCBI, GEO database (Accession No: GSE36376 and GSE45436). The comparative expression values were showed as scatter plots. The median expression is indicated by horizontal line. The microRNA expression levels are shown on the *y* axis (log2 intensity, ***P<0.001, Student's *t* test). (B) Western blot analysis. SNU-449 and SKHep-1 cells were transfected with miR-31 mimics after transfected with *Dicer* siRNA or negative control siRNA (N.C). The protein levels of HDAC2, CDK2 and Dicer were detected with their specific antibodies. GAPDH was used as the endogenous loading control. (C) The target sites of miR-31 in 3′-UTR of *HDAC2* and *CDK2* are shown as a schematic representation. The target sequence was predicted by miRNA target prediction program, miRWalk (http://www.umm.uni-heidelberg.de/apps/zmf/mirwalk/). (D) Luciferase reporter assay. Wild type or mutant 3′-UTR construct of *HDAC2* and *CDK2* were cloned into a psi-CHECK2 vector, respectively, and co-transfected with miR-31 mimics in SNU-449 and SKHep-1 cells. Renilla luciferase activities were normalized to firefly luciferase activities. All assays were performed in triplicates and repeated at least three times. (means ± SD; *P<0.05; **P<0.005, ***P<0.001, Student's *t* test). (E) The Biotin-labeled miR-31-mRNA pull down assay. SNU-449 and SKHep-1 cells were transfected with Biotin-labeled microRNA control (Bio-N.C) or Biotin-labeled miR-31 mimics for 48 hours. The expressions of *HDAC2* and *CDK2* were measured by qRT-PCR and normalized to *GAPDH* (means ± SD; **P<0.005; ***P<0.001).

It has been demonstrated that all the known processes of cancer biology, including apoptosis, proliferation, survival, and metastasis, are regulated by small regulatory non-coding RNAs consisting of approximately 19–25 nucleotides; e.g. miRNAs [5]. Therefore, we hypothesized that some cancer-driver genes targeted by miR-31 are up-regulated in HCC as miR-31 was down-regulated in HCC. Thus, to identify miR-31 target genes, we used the target prediction program, miRWALK (http://www.umm.uniheidelberg.de/apps/zmf/mirwalk/), a comprehensive database on miRNAs with eight established program (RNA22, miRanda, miRDB, TargetScan, RNAhybrid, PITA, PICTAR, and Diana-microT) [16]. From this database, at least in six out of eight different prediction programs, 399 genes were predicted to be targeted by miR-31 (data not shown). Of these 399 genes, we were able to identify 36 genes that were commonly up-regulated in three different HCC cohort data sets, GSE14520, GSE22058 and GSE16757, respectively (Supplementary Table S1). Among these, our previous study has demonstrated that histone deacetylase 2 (HDAC2) and cyclin-dependent kinase 2 (CDK2) were overexpressed in HCC [17]. We then recapitulated the expression of *HDAC2* and *CDK2* genes from two more cohorts of HCC patients to generalize our finding. Consistently, *HDAC2* and *CDK2* genes were significantly over-expressed in these two different HCC cohorts (Fig. 2A). The fact that *HDAC2* and *CDK2* are up-regulated in HCC led us to hypothesize that normal *HDAC2* and *CDK2* expressions are balanced by endogenous miR-31, which selectively controls *HDAC2* and *CDK2* mRNA translation in normal hepatic liver cells. Thus, to support our hypothesis that HDAC2 and CDK2 expressions are regulated by miR-31 in HCC cell lines, we introduced *Dicer* specific siRNAs to block miRNA biogenesis in HCC cells. As shown in Fig. 2B, *Dicer* knockdown augmented HDAC2 and CDK2 protein expressions in SNU-449 and SKHep-1 cells, whereas co-transfection of miR-31 mimics attenuated *Dicer* knockdown effect on the same cells. Then, to determine whether HDAC2 and CDK2 are selectively regulated by miR-31 via direct interaction with the 3′-UTR of these genes, we cloned the 3′-UTR of *HDAC2* and *CDK2* into a reporter vector linking the luciferase open reading frame downstream to generate psi-CHECK2-HDAC2_3′-UTR and psiCHECK-CDK2_3′-UTR plasmid, respectively (Fig. 2C and Supplementary Fig. S1). Next, to verify that miR-31 specifically binds to 3′UTRs of *CDK2* and *HDAC2* to interfere translation of those transcripts, mutant vectors harboring random mutation sequences of miR-31 biding sites of the 3′UTR of *CDK2* and *HDAC2* genes were generated, and then each vector was co-transfected with miR-31 into SNU-449 and SKHep-1 cells. It was found that miR-31 was able to suppress reporter gene activity in these cells, whereas mutants plasmids showed no changes in the reporter gene activity in both SNU-449 and SKHep-1 cells indicating miR-31 selectively regulate both HDAC2 and CDK2 expressions in HCC cells *in vitro* (Fig. 2D). In addition, to clarify the direct interaction between miR-31 and 3′-UTRs of the two transcripts, we carried out biotin-labeled RNA pull-down assays. As expected, when Bio-miR-31 mimics were transfected to both SNU-449 and SKHep-1 cells, *HDAC2* and *CDK2* transcripts were enriched in these cells compared to that of Bio-microRNA control transfectants (Fig. 2E). These results demonstrate that miR-31 is a direct regulator of endogenous expression HDAC2 and CDK2 in liver cancer cells.

**Figure 3:**
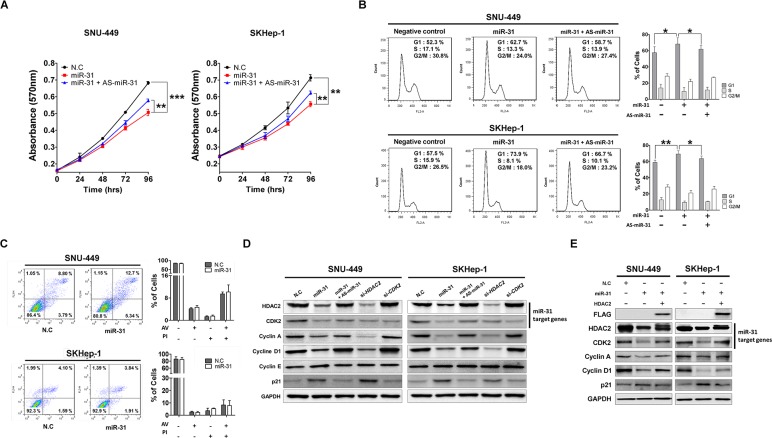
MiR-31 inhibits liver cancer cell growth by targeting G1/S transition regulatory molecules (A) Ectopic expression of miR-31 suppressed SNU-449 and SKHep-1 cell proliferation. Transfection of antisense miR-31 (AS-miR-31) attenuated anti-growth effect of miR-31. The cell viability was determined by measuring MTT absorbance at A570. Cell growth was measured at every 24 hours. N.C represents negative control microRNA (means ± SD; **P<0.005, ***P<0.001 compared to control, Student's *t* test). (B) After transfection of miR-31 mimics or co-transfection with AS-miR-31 to SNU-449 and SKHep-1, the DNA content of PI-stained cells was analyzed by flow-cytometry (means ± SD; **P<0.005, ***P<0.001 compared to control, Student's *t* test). The stained cell number ratios are presented in bar graph (means ± SD; *P<0.05, **P<0.005, Student's *t* test). (C) Evaluation of apoptosis by annexin V-PITC (AV) and propidium iodide (PI) staining and analysis by flow-cytometry in SNU-449 and SKHep-1 cells after transfection of miR-31 mimics or negative control miRNA (N.C). (D) SNU-449 and SKHep-1 cells were transfected with miR-31 mimics or co-transfected with AS-miR-31. si-*HDAC2* and si-*CDK2* were used for knockdown of miR-31 target genes, respectively. The protein expression levels of G1/S regulatory molecules were analyzed by immunoblotting. N.C represents negative control miRNA. (E) Co-transfection of miR-31 with 3′UTR-deleted *HDAC2* plasmid (pME18s-HDAC2-FLAG) rescued the expressions of G1/S regulatory molecules. The expressions were analyzed by immunoblotting.

Next, to investigate biological functions of miR-31 in hepatocellular malignant proliferation and transformation, we attempted ectopic expression of miR-31 and studied in the 3-(4, 5-dimethylthiazol-2-yl)-2, 5-diphenytetrazolium bromide (MTT) assay for the measurement of cell growth rate of two different liver cancer cell lines, SNU-449 and SKHep-1. Ectopic overexpression of miR-31 resulted in reduced growth rates of these two different liver cancer cell lines, whereas co-transfection with AS-miR-31 (an antisense inhibitor of miR-31) significantly blocked this anti-growth effect (Fig. 3A). In contrast, overexpression of miR-31 in MIHA and L-O2 (immortalized normal hepatic cell lines) did not effect on cell growth rates of these two different cell lines (Supplementary Fig. S2). The anti-growth effect could be partially explained by the disruption of cell growth regulation on miR-31 targeting, such as cell cycle arrest, cellular senescence or apoptosis. Thus, we next explored the effects of miR-31 overexpression on cell death and cell cycle regulation. Flow cytometric cell cycle analysis indicated that miR-31 overexpression led to an increase in the number of cells in the G1 phase with a concomitant decrease in the number of cells in the S phase and G2/M phase, but AS-miR-31 co-transfection attenuated this effect in the same cells (Fig. 3B). We then stained the cells with annexin V-FITC and PI after transfection of miR-31 mimics for apoptosis analysis. However, miR-31 overexpression showed no significant induction of apoptotic cells compared to miRNA control (Fig. 3C).

Next, to better understand the underlying mechanism of the growth inhibition elicited by miR-31, western blot analysis was performed for cell cycle regulatory proteins and miR-31-targeting molecules, HDAC2 and CDK2. Our previous studies have suggested the oncogenic potential of HDAC2 overexpression in *in vitro* and *in vivo* liver tumorigenesis [17, 18]. In G1/S transition, targeted-disruption of HDAC2 selectively induced the expression of negative cell cycle regulators, p16^INK4A^ and p21^WAF1/Cip1^, and simultaneously suppressed the expression of cell cycle components, such as cyclin D1, CDK4 and CDK2. In our study, western blot analysis showed that HDAC2 and CDK2 protein levels were decreased after ectopic expression of miR-31, and simultaneously induced p21^WAF1/Cip1^ and suppressed the expression of cyclin A and cyclin D in both SNU-449 and SKHep-1 cells. In contrast, this result was significantly attenuated by the co-transfection of AS-miR-31 in the same cells (Fig. 3D). Notably, we also observed that targeted-disruption of HDAC2 recapitulated the effect of ectopic miR-31 expression on the same molecules whereas *CDK2* knockdown did not affect. Although we showed that ectopic miR-31 elicited inhibition of cellular growth of liver cancer cells through targeting HDAC2, it is necessary to prove that ectopic overexpression of 3′ UTR-deleted *HDAC2* plasmid (pME18s-HDAC2-FLAG) can rescue the effects on cell cycle molecules in the same cells. As expected, ectopic overexpression of HDAC2 protein remarkably rescued the expression of cyclin A and D1 and simultaneously suppressed p21^WAF1/Cip1^ to basal level in the same cells (Fig. 3E). These results demonstrate that miR-31 regulates cell cycle molecules through the selective control of HDAC2 expression in liver cancer cells.

### MiR-31 regulates metastatic potential of liver cancer cells and is suppressed by deregulation of epigenetic modifiers

**Figure 4:**
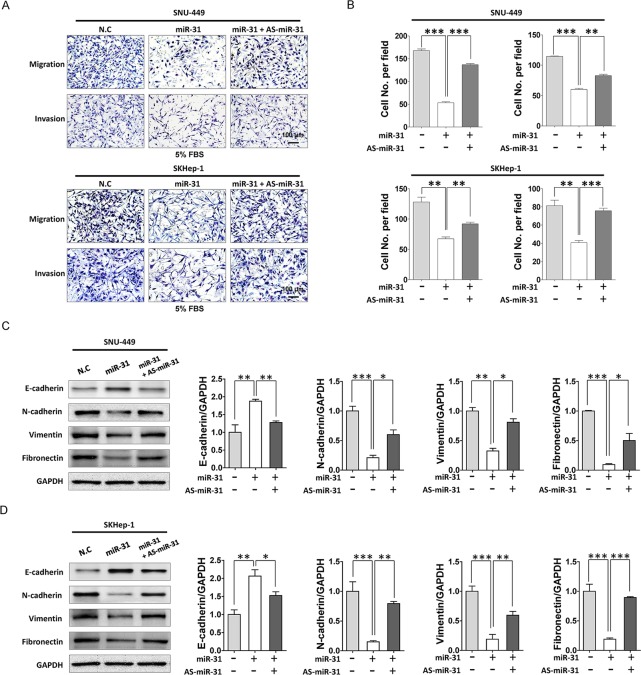
MiR-31 suppressed motility and invasion of HCC cells (A) Motility and invasion assay of liver cancer cells transfected with miR-31 or co-transfected miR-31 with AS-miR-31. Invasion assay was performed with transwell-inserts coated with Matrigel. Images were taken with invert microscope (magnification, x100). (B) The cell number of migrated cells were counted in randomly selected fields and presented in bar graph (means ± SD; *P<0.05; **P<0.005; ***P<0.001, Student's *t* test). (C and D) Western blot analysis of E-cadherin, N-cadherin, vimentin and fibronectin of SNU-449 (C) and SKHep-1 (D) cells. GAPDH was used as the endogenous loading control. Quantitative analysis of western blot data. Densitometry was used to quantify western blot data for E-cadherin, N-cadherin, vimentin and fibronectin, respectively (means ± SD; *P<0.05; **P<0.005; ***P<0.001, Student's *t* test).

**Figure 5:**
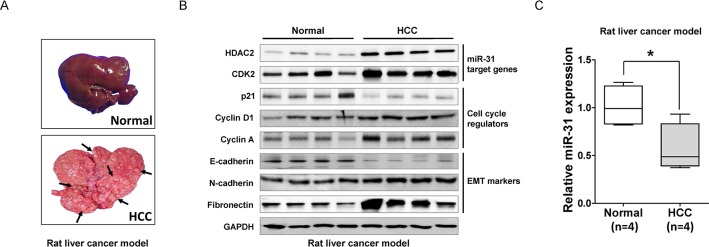
*In vivo* validation of miR-31 regulating molecules in DEN-induced rat liver cancer model (A) Macroscopic observation of the whole liver from the DEN-induced rat model (arrows; tumors). (B) Western blot analysis. The direct target molecules of miR-31, HDAC2 and CDK2, as well as the cell cycle regulators and EMT markers, were analyzed with immunoblotting. (C) A qRT-PCR analysis of miR-31 expression in DEN-induced rat HCC model (means ± SD; *P<0.05, Student's *t* test).

EMT has been proposed as a key process in cancer progression. Tumor cells that acquired EMT and metastatic ability show mesenchymal, fibroblast-like phenotypes with cell-cell contact, polarity loss, enabling cells migration and invasion [19]. MiR-31 is well known metastatic suppressor by direct targeting integrin family, RhoA and RDX in various cancers but unknown in liver cancer [14]. Thus, to elucidate the role of miR-31 in the malignant behavior of liver cancer cells, we performed *in vitro* motility and invasion assays. A modified Boyden chamber assays revealed that ectopic expression of miR-31 mimics significantly suppressed chemoattractant (5% fetal bovine serum)-stimulated migratory and invasive responses of both SNU-449 and SKHep-1 cells, whereas AS-miR-31 co-transfection significantly rescued anti-migratory and invasion effects in the same cells (Fig. 4A and B). In similar, when *ras*-transformed NIH-3T3 cells were transfected with miR-31 mimics to generalize the effect of miR-31 in the regulation of metastatic potential, we obtained consistent results in both motility and invasion assays (Supplementary Fig. S3). To gain further insight into the regulatory effect of miR-31 on EMT, western blot analysis was performed for the EMT regulatory proteins in in liver cancer cells. Notably, N-cadherin, vimentin and fibronectin, hallmarks of EMT, were dramatically decreased in miR-31 mimics transfectants, whereas E-cadherin, an epithelial markers, was increased in both SNU-449 and SKHep-1 cells (Fig. 4C and D). In contrast, the suppressive effect of miR-31 on EMT molecules in liver cancer cells was significantly rescued by the con-transfection of AS-miR-31. These results suggest that anti-metastatic potential of miR-31 could be attributed to the selective regulation of EMT proteins in liver cancer cells. Next, to validate our observations in *in vivo* model, we prepared diethylnitrosamine (DEN)–induced liver cancer rat models and examined cell cycle molecules and HDAC2 expressions in hepatocellular carcinoma tissues (Fig. 5A). DEN-induced rat HCC tissues showed the upregulated expression levels of HDAC2, CDK2, cyclin D1, cyclin A, N-cadherin, fibronectin and suppressed expression levels of p21^WAF1/Cip1^ and E-cadherin accompanied by reduced-miR31 expression compared to normal rat hepatic tissues (Fig. 5B and C).

**Figure 6:**
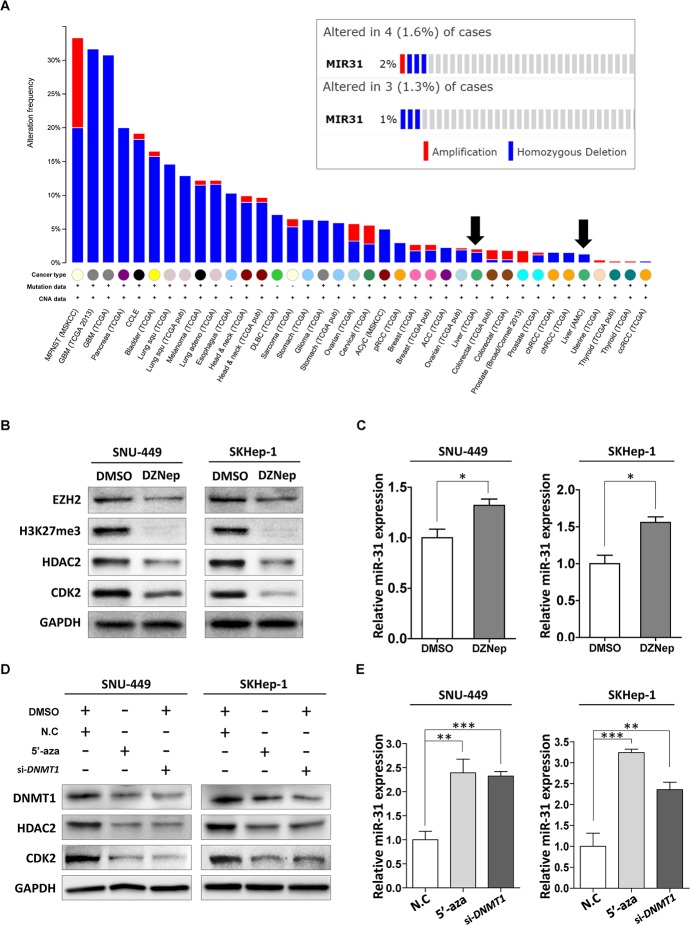
Inactivation mechanism of tumor suppressor miR-31 in liver cancer (A) Cross-cancer summary of homozygous mutations and copy number variations in all cancers available on cBioPortal (http://www.cbioportal.org). The arrows represents hepatocellular carcinoma. (B) Western blot analysis. SNU-449 and SKHep-1 cells were treated with DZNep (0.1% DMSO or 5 μM of 5-zaz-dC). The expressions of EZH2, H3 Lys-27 hyper-methylation (H3K27me3), HDAC2 and CDK2 were analyzed with immunoblotting. (C) A qRT-PCR analysis of miR-31 expression in DZNep treated liver cancer cell lines (means ± SD, *P<0.05, Student's *t* test). (D) Western blot analysis. The liver cancer cell lines, SNU-449 and SKHep-1, were treated with indicated drug (01% DMSO or 10 μM of 5-aza-dC), or transfected with siRNA (negative control siRNA, si-*DNMT1*), and analyzed protein expressions of miR-31 target genes, HDAC2 and CDK2. (E) A qRT-PCR analysis of miR-31 in 5-aza-dC treated, or siRNA (negative control siRNA, si-*DNMT1*) transfected SNU-449 and SKHep-1 cells (means ± SD; **P<0.005; ***P<0.001, Student's *t* test).

Lastly, to gain further insight into the inactivation mechanism of miR-31 in liver cancer, we investigated whether miR-31 locus is deleted in liver cancer using TCGA data available on cBioPortal (www.cbioportal.org). From this, we found that the incidence of homozygous deletion of miR-31 gene locus was very low (1.3 ~ 1.6%) in liver cancer compared to that of bladder or pancreatic cancer (Fig. 6A). In addition to genomic deletion, epigenetic gene silencing is another tumor suppressor inactivation mechanism. Since EZH2, a core component of polycomb repressive complex2 (PRC2), was reported to be over-expressed in HCC, we assumed that hyper-methylation of H3 Lys-27 residue may be related with the suppression of miR-31 [20]. To clarify that expression of miR-31 is regulated by EZH2, cells were treated with DZNep (3-Deazaneplanocin A, an inhibitor of S-adenosylmethionine-dependent methyltransferase, and stimulates degradation of EZH2). Notably, treatment of DZNep elicited remarkable suppression of EZH2, HDAC2 and CDK2 proteins with concomitant increase of miR-31 expression in SNU-449 and SKHep-1 cells (Fig. 6B and C). Promoter hyper-methylation is also another efficient way of gene deregulation. In prostate cancer, hyper-methylation of miR-31 promoter was responsible for its low expression and contributed tumorigenesis [21]. Therefore, cells were treated with 5-aza-dC (Azacitidine, 5-aza-2′deoxycytidine), a chemical analogue of the cytosine nucleoside causing hypomethylation of DNA, or DNMT1 (DNA-methyltrasnferase 1)-siRNA, and performed western blot analysis. Disruption of DNA methylation by either 5-aza-dC treatment or DNMT1 knockdown caused the induction of miR-31 expression, and thereby suppressed HDAC2 and CDK2 expression in both SNU-449 and SKHep-1cells (Fig. 6D and E). These results provide the underlying mechanisms leading to the suppression of endogenous miR-31 in HCC.

## DISCUSSION

To date, many studies have shown that miRNAs exhibit altered expression levels in various cancers and may play potential roles as diagnostic and prognostic biomarkers of cancers. MiR-31 is one of well identified miRNAs in cancer biology, and interestingly, regulation patterns and functions of miR-31 were diverse depending on cancer types. Thus, it is assumed that miR-31 has a specific function in each type of malignancy, and several mechanisms, including methylation-dependent silencing and local deletion, may explain its different roles in different tumor types. However, little is known about the miR-31 status in patients with HCC and the possible roles in hepatocarcinogenesis. In the present study, we demonstrated that miR-31 functions as a tumor suppressor in the development of HCC by negative regulation of the major components in the cell cycle transition and EMT processing of cancer cells.

In previous studies, miR-31 was reported as an oncomir in several human cancers. For example, miR-31 was demonstrated that overexpression of miR-31 led to increased growth rate by targeting suppressors, *LATS2* and *PPP2R2A* in lung cancer [22]. Ectopic expression of miR-31 increased the oncogenic potential of head and neck squamous cell carcinoma cells under normoxic conditions in cell culture or tumor xenografts by impeding factor-inhibiting hypoxia-inducible factor [23]. In colorectal cancer, it was demonstrated that miR-31 plays a significant role in activating the RAS signaling pathway through the inhibition of RASA1 translation, thereby improving colorectal cancer cell growth and stimulating tumorigenesis [24]. On the contrary to this, miR-31 was also reported as a tumor suppressor in other type of cancers. In breast cancer, miR-31 was under expressed in metastatic tumor and negatively correlated with high risk of metastasis. To be specific, ectopic miR-31 repressed metastatic potential and this result was explained by miR-31-mediated repression of *ITGA5*, *RDX* and *RhoA* [10]. In addition to breast cancer, not only that miR-31 acted as a metastatic suppressor by regulating these genes, but also elicited cell cycle arrest and apoptosis in lung cancer [25]. In another study, it was identified that *WAVE3*, actin cytoskeleton regulating molecule, was shown to be directly regulated by miR-31 [26]. Expression of miR-31 correlates inversely with breast cancer progression in humans, where an increase in expression of miR-31 target genes was observed as tumors progressed to more aggressive forms. Furthermore, in prostate cancer, miR-31 was identified to negatively regulate *E2F1*, *E2F2*, *EXO1*, *FOXM1*, and *MCM2*, which are the key regulatory proteins in cell cycle regulation, and thereby demonstrated that the downregulation of miR-31 disrupts cellular homeostasis and contributes to the evolution and progression of prostate cancer. [21].

For liver cancer, one recent study reported that miR-21, miR-31, miR-122, miR-221, miR-222 were significantly up-regulated in HCC tissues, whereas miR-145, miR-146a, miR-200c, and miR-223 were found to be down-regulated [15]. However, authors concluded that high level of miR-21, miR-31, miR-122, and miR-221 expression was correlated with cirrhosis but only miR-21 and miR-221 were associated with tumor stage. Thus, the aberrant expression of these miRNAs, such as miR-31 and -122 remained to be validated in liver cancer. Our data, contradictive with previous observation, indicated that miR-31 expression was significantly down-regulated in patients with HCC. Notably we also able to generalize the repressive status of miR-31 expression in HCC by recapitulating miR-31 expression from the various large cohorts of HCC patients that are available from the NCBI, GEO database (Fig. 1). From this, obviously miR-31 is appeared to be suppressed in HCC compared with that of non-cancerous surrounding tissues. In many different types of cancers, repressed miR-31 expression was demonstrated to contribute malignant transformation and proliferation of cancer cells. Earlier report showed that miR-31 is located at the chromosome 9q21.3, and this locus is very close to the locations of tumor suppressors, *CDKN2A* and *CDKN2B*, that is frequently deleted in many cases of cancers, which may result in down-regulation of miR-31 [27, 28]. However, our investigation found that the incidence of homozygous deletion of miR-31 gene locus was very low (1.3 ~ 1.6%) in liver cancer compared to other cancers, bladder or pancreatic cancer, that exhibiting high incidence of homozygous deletion (Fig. 6A). On the other hand, hyper-methylation of miR-31 promoter region was also reported as inactivating mechanism for miR-31 in prostate cancer [21]. Our results showed inhibition of DNA methylation by either 5-aza-dC treatment or knockdown of DNMT1 caused the induction of miR-31 expression, and consequently suppressed HDAC2 and CDK2 expression in liver cancer cells. Notably we also found that treatment of DZNep elicited remarkable suppression of EZH2, HDAC2 and CDK2 proteins with concomitant increase of miR-31 expression in liver cancer cells (Fig. 6B-E). Although the underlying mechanism leading to the suppression of miR-31 should be clearly elucidated, these epigenetic alterations cause the down-regulation of miR-31 and thereby contribute to the hepatocellular malignant transformation and proliferation.

As a tumor suppressive role of miR-31 in tumorigenesis, it was reported that miR-31 directly or indirectly controls expressions of specific proteins involving hallmarks of cancers, such as cell cycle, apoptosis, migration and cytoskeleton regulating molecules. Our results showed that ectopic expression of miR-31 resulted in suppression of HDAC2 and CDK2 protein expression in liver cancer cells. Our recent study has demonstrated that inactivation of HDAC2 resulted in induction of p16^INK4A^ and p21^WAF1/Cip1^ while positive cell cycle regulators, such as cyclin D1, CDK4 and CDK2 are simultaneously suppressed [17]. Our results demonstrated that repression of miR-31 contributes to transcriptional activation of HDAC2, and, thereby causes the acceleration of cell cycle transition of cancer cells through selective regulation of cell cycle components (Fig. 3). Previous study has also demonstrated that the knockdown of HDAC2 significantly decreased metastatic potential in HCC cells, and HDAC2 expression was highly up-regulated in patients with HCC with vascular invasion [18]. In parallel with our previous observation, miR-31 selectively regulated EMT proteins, N-cadherin, E-cadherin, vimentin and fibronectin, to control metastatic potential of liver cancer cells (Fig. 4). Interestingly when we performed prediction analysis of putative miR-31 targets by using miRWALK database, 399 genes were resulted in possible targets of miR-31. However, N-cadherin, E-cadherin, vimentin and fibronectin were not found as miR-31 target genes (data not shown). Thus, suppressive function of miR-31 on EMT molecules seems to be indirect effect of miR-31, and HDAC2 may also contribute to selective regulation of these EMT molecules.

Taken together, we present evidences that miR-31 functions as a tumor suppressive miRNA by directly regulating HDAC2 and CDK2 expression in liver cancer progression. MiR-31 was significantly down-regulated in overt HCC and ectopic expression of miR-31 mimics inhibited *in vitro* tumor growth and metastatic ability in liver cancer cell lines. Although further research is required to identify regulatory mechanisms for the repression of miR-31 in liver cancer, the results suggest that the miR-31 may play a central role in hepatocellular malignant transformation and proliferation providing novel therapeutic intervention of liver malignancy.

## MATERIALS AND METHODS

### Tissue samples

Total 9 HCC tissues with their corresponding normal tissues were obtained from Yonsei University, School of Medicine (Seoul, Korea). Subjects were informed from the study design and purpose according to the Declaration of Helsinki. Written informed consent was obtained from all subjects, and the study was approved by the institutional review board at the Catholic University of Korea (IRB approval number; MC12EISI0106).

### Cell culture

The human HCC cell lines, HepG2, Hep3B, Huh7, PLC/PRF/5, SKHep-1, SNU-182 and SNU-449 were obtained from KCLB (Korean Cell Line Bank, Seoul, South Korea). The liver cell lines, MIHA and L-O2 were purchased from ATCC (Manassas, VA, USA). Each cell line was maintained in RPMI-1640 or DMEM medium (Lonza, Walkersville, MD) supplemented with 10% fetal bovine serum (Sigma, St. Louis, MO) and 100 units/ml of penicillin–streptomycin (Invitrogen, Carlsbad, CA). All cells were cultured at 37 °C in a humidified incubator with 5% CO_2_. To inhibit global histone methylation and DNA methylation, DZNep (10 μM, Sigma-Aldrich, St. Louis, MO, USA) and 5-aza-dC (5 μM, Sigma-Aldrich, St. Louis, MO, USA) were treated.

### Transfections

Small interfering RNAs (siRNAs) of *Dicer* (sense: 5′-UAAAGUAGCUGGAAUGAUG-3′, antisense: 5′-CAUCAUUCCAGCUACUUUA-3′), *CDK2* (sense: 5′-GGAGCUUGUUAUCGCAAAU, antisense: 5′-AUUUGCGAUAACAAGCUCC-3′), *DNMT1* (sense: 5′-CACUGGUUCUGCGCUGGGA-3′, antisense: 5′-UCCCAGCGAGAACCAGUG-3′) and microRNA mimics of miR-31 (sense: 5′-AGGCAAGAUCUGGCAUAGCU-3′, antisense: 5′-AGCUAUGCCAGAUCUUGCCU-3′) were synthesized by Genolution (Seoul, Korea). Peptide nucleic acid-modified miRNA inhibitors were purchased from PANAGENE (Daejeon, Korea). Negative control RNA duplexes was purchased from Ambion (Austin, TX). Negative control siRNA or miRNA is abbreviated as ‘N.C' in. Transfections were carried out using lipofectamine 2000 and lipofectamine RNAiMAX (Invitrogen, Carlsbad, CA), according to the manufacturer's instructions.

### Quantitative real time-polymerase chain reaction (qRT-PCR)

Total RNA was isolated from frozen tissues and cell lines with Qiazol reagent and miRNeasy mini kit (Qiagen). The TaqMan microRNA reverse transcription kit (Applied Biosystems, Carlsbad, CA) was used to synthesize the cDNA for specific miRNA. The qRT-PCR reactions were performed with iTaq™ Universal SYBR® Green supermix (Bio-Rad, Hercules, CA) and monitored in real-time by an iQ™-5 (Bio-Rad). The primers for amplification of miR-31 are 5′-TAATACTGCCTGGTAATGATGA-3′ and 5′-GTCGTATCCAGTGCAGGGTCCGAGGTATTC GCACTGGATACGACAGCTAT-3′. The expression level of U6 snRNA (forward: 5′-GCGCGTCGTGAAGCGTTC-3′, reverse: 5′-GTGCAGGGTCCGAGGT-3′) was used as an internal control for normalization.

### Western blotting

Cells were lysed in protein extraction buffer (50 mM HEPES, 5 mM EDTA, 50 mM NaCl, 1% Triton X-100, 50 mM NaF, 10 mM Na_2_P_2_O_7_, 1 mM Na_3_VO_4_, 5 μg/mL aprotinin, 5 μg/mL leupeptin, 1 mM PMSF, and protease inhibitor cocktail). Lysates containing equal amounts of proteins were separated by SDS–PAGE and transferred onto polyvinylidene difluoride membrane (Bio-Rad, Hercules, CA). The blots were blocked with a 5% skim milk solution and incubated with the following antibodies: anti-Dicer, anti-cyclin A, anti-cyclin D1, anti-cyclin E, anti-p21^WAF1/CIP1^, anti-EZH2,(Cell Signaling Technology, Danvers, MA), anti-HDAC2, anti-GAPDH and anti-CDK2 (Santa Cruz Biotechnology, Santa Cruz, CA), anti-N-cadherin, anti-E-cadherin, anti-vimentin and anti-fibronectin (BD Transduction, San Jose, CA), anti-DNMT1 (Abcam, Cambridge, MA) and anti-H3K27me3 (Millipore, Billerica, MA). The Immobilon™ Western blot detection system (Millipore, Billerica MA) was used to detect bound antibodies. The intensities of the Western blot bands were quantified using LAS 3000 (Fuji Photo Film Co., Japan).

### Cell proliferation assay

3-(4, 5-Dimethylthiazol-2-yl)-2, 5-diphenyltetrazoliumbromide (MTT) assays were conducted to measure the relative number of viable cells. At indicated time points, medium was replaced with the fresh medium supplemented with MTT (0.5 mg/ml) (Sigma-Aldrich, St. Louis, MO). Absorbance was measured using a multilabel plate reader (VICTOR3TM, PerkinElmer, Bridgeport Avenue Shelton, CT,) at a wavelength of 570 nm.

### Flow cytometry analysis

Cells were transfected with microRNAs for 6 hours. After 48hours of incubation in RPMI-1640 complete medium, the cells were trypsinized and washed with cold NaCl/P_i_, The cells were fixed in pre-chilled 70% ethanol at 20 °C overnight. For measurement of DNA content, cells were stained with propidium iodide solution (50 ug/ml propidium iodide, 100 ug/mL RNase A, 0.05% Triton X-100 in NaCl/P_i_), and incubated at 37 °C in the dark for 30 min. DNA content was examined by flow cytometry using a FACSCalibur (BD Biosciences, San Jose, CA) with FLOWJO software (Tree Star, Ashland, OR).

### Motility and invasion assay

For *in vitro* cell motility and invasion assay, Transwell plates and cell culture inserts (BD Biosciences, San Jose, CA) were used. For the coating of invasion assay, Matrigel (BD Biosciences, San Jose, CA) was diluted to 0.3 mg/ml concentration with Coating buffer (0.01 M Tris, 0.7% NaCl, pH 8.0) and 100 μl Matrigel was coated onto upper compartment of cell culture insert. After incubation for 1 h at 37 °C, the cell culture insert was ready for seeding. After transfection of miR-31, SNU-449 and SKHep-1 cells were appropriately (5 × 10^4^ cell/well for motility assay, 1 × 10^5^ cell/well for invasion assay) seeded into the cell culture insert with serum-free media and 5% fetal bovine serum was used as a chemoattractant. After 4 h (motility) or 12 h (invasion) of incubation at 37 °C, migrated or invaded cells were stained using Diff-Quik staining kit (Sysmex, Japan). The images of cells were photographed with Axiovert 200 inverted microscope (Zeiss, Germany) at ×200 magnification and the cell number was counted in three random fields of view.

### *HDAC2* and *CDK2* 3′UTR constructs

Cloning method for psi-CHECK2-HDAC2-3′UTR has previously been described [29]. CDK2 3′-UTR region were amplified by PCR with liver cancer cell line cDNA using following primers: 5′-ATAAGAATGCGGCCGCTAAACTATTAGCC TTCTTGAAGCCCCC-3′ (forward), 5′-CCGCTCGAGCGGTGAACTATAAAACTAGG CACATTTTTT-3′ (reverse). The PCR product was cloned into the Xho I/Not I site of a psiCHECK-2 vector (Promega, Madison, WI). For mutagenesis of the miR-31-binding site, a QuickChange site-directed Mutagenesis Kit (Agilent Technologies, Palo Alto, CA) was used according to the manufacturer's instructions. Primers for mutagenesis were synthesized as following sequences: HDAC2_3′UTR_mut forward: 5′-ACTTGTATTGACCAGACAAGGCAATA-3′, HDAC2_3′UTR_mut reverse: 5′-TATTGCCATGTCTGGTCAATACAAGT-5′ and CDK2_3′UTR_mut forward: 5′-TACCCTAGTTAGTGTTTAGGCACA-3′, CDK2_3′UTR_mut reverse: 5′-TATTGCCATGTCTGGTCAATACAAGT-3′ (reverse).

### Luciferase activity assay

Cells were seeded into 12-well plates one day before transfection. After 16 hr of plating, the cells were co-transfected with 200 ng of psiCHECK-2 plasmids and 100 nM of miRNA. After 48 h, luciferase activities were measured with Dual-Luciferase Reporter Assay system (Promega, Madison, WI). Renilla luciferase activity was normalized to firefly luciferase activity.

### Biotin-labelled RNA pull down assay

SNU449 and SKHep-1 cells were transfected with Bio-miR-31 or Bio-miR-control in two 60mm dishes. After 48 hours of incubation, the cells were trypsinized and washed twice with PBS. Cells were resuspended in 0.7 ml of lysis buffer (20 mM Tris (pH 7.5), 100 mM KCl, 5 mM MgCl_2_, 0.3% IGEPAL CA-630) and then incubated on ice for 20 min. The cytoplasmic lysate was isolated by centrifugation at 10,000x for 15 min and supernatant was collected. The lysate was added to the Strepatavidin-coated magnetic beads (Invitrogen) and incubated and incubated overnight at 4°C. The beads were washed with lysis buffer for 5 times and 100ul of lysis buffer with DNaseI (2U/ul) was added. After incubation at 37°C for 10 min, lysates were centrifuged at 5,000g for 5 min and the supernatant was discarded. Protein kinase K (20mg/ml) and 1ul of 10% SDS in 100 ul of lysis buffer were added to the pellet and incubated at 55°C for 20 min. RNA bound to the beads (pull-down RNA) or from 10% of the extract (input RNA), was isolated with Trizol reagent (Invitrogen). The levels of HDAC2 and CDK2 in the Bio-miR-31 pull-down were quantified by qRT-PCR. GAPDH was used for normalization.

### Rat tumor model xenograft assay

To induce HCC in rat, diethylnitrosamine (DEN) was used as previously described [30, 31].

### Statistical analysis

All experiments were performed at least three times, and all samples were analyzed in triplicates. Results were presented as mean ± standard deviation (SD). Statistical difference between each group was assessed by unpaired two-tailed Student *t*-test using Graphpad^TM^ 5.0 software. *P*-values <0.05 were considered statistically significant.

## SUPPLEMENTARY MATERIAL, FIGURES AND TABLE


