# Supplementary material for: MicroRNA-31 functions as a tumor suppressor by regulating cell cycle and epithelial-mesenchymal transition regulatory proteins in liver cancer
Source: Oncotarget. 2015 Mar 10;6(10):8089–102. doi: 10.18632/oncotarget.3512 (PMC4480737; doi:10.18632/oncotarget.3512)
Supplement: Supplementary file 1 [file oncotarget-06-8089-s001.pdf]

MicroRNA-31 functions as a tumor suppressor by regulating cell cycle and epithelial-mesenchymal transition regulatory proteins in liver cancer

Supplementary Material

Supplementary Table S1: Up-regulated genes predictively targeted by miR-31 in HCC

Gene symbol	Fold change		
	GSE14520	GSE22058	GSE16757
MYB	1.382	1.944	1.15
WHSC1	2.185	1.334	1.205
AURKB	1.503	4.92	2.111
ARPC5	2.016	1.315	1.5
SSH1	1.257	1.352	1.266
ITGAV	1.882	1.128	1.818
ARHGEF2	1.368	1.376	1.345
C1orf2	1.861	3.006	1.508
HYOU1	1.781	1.355	1.748
PPP2R5A	1.831	1.57	1.457
CHEK1	1.524	3.872	1.738
DNMT3B	1.398	4.079	1.233
LASP1	1.707	1.424	1.605
SRPK1	1.986	1.443	1.49
**HDAC2**	1.772	1.33	1.335
KCMF1	1.339	1.331	1.17
FOXM1	2.262	9.644	1.677
CTNND2	1.419	1.909	1.311
SEMA5B	N.A	3.57	1.216
NDRG3	1.285	1.527	1.495
CDC23	2.208	1.594	1.337
ARPP-19	1.56	N.A	1.394
CHD7	2.143	1.594	1.381
RRM2	6.073	2.099	N.A
CCNB1	8.319	2.138	N.A
IQGAP3	N.A	3.131	4.929
PARP1	2.217	1.788	1.833
CDT1	1.155	1.804	2.419
CDC25B	1.812	1.837	1.001
**CDK2**	1.579	1.319	1.321

E2F1	1.148	4.99	1.892
UBXD8	1.334	1.236	1.117
CIAPIN1	1.479	N.A	1.237
ITPA	1.382	1.319	1.15
TFRC	2.825	1.735	1.367
YKT6	1.387	1.449	N.A

"N.A", Not available

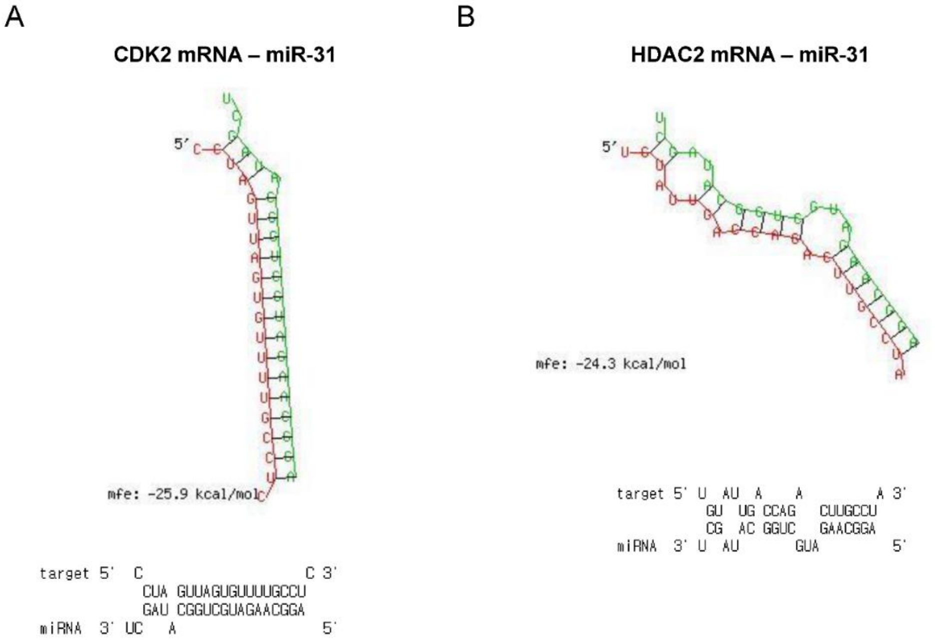

Supplementary Figure S1: The hybrid models between miR-31 and its target transcripts (A and B) Calculated miR-31 and 3'-UTRs of CDK2 and HDAC2 hybrid structures. CDK2 and HDAC2 were predicted with RNAhybrid (http://bibiserv.techfak.uni-bielefeld.de/rnahybrid/). MFE represents the calculated minimal free energy.

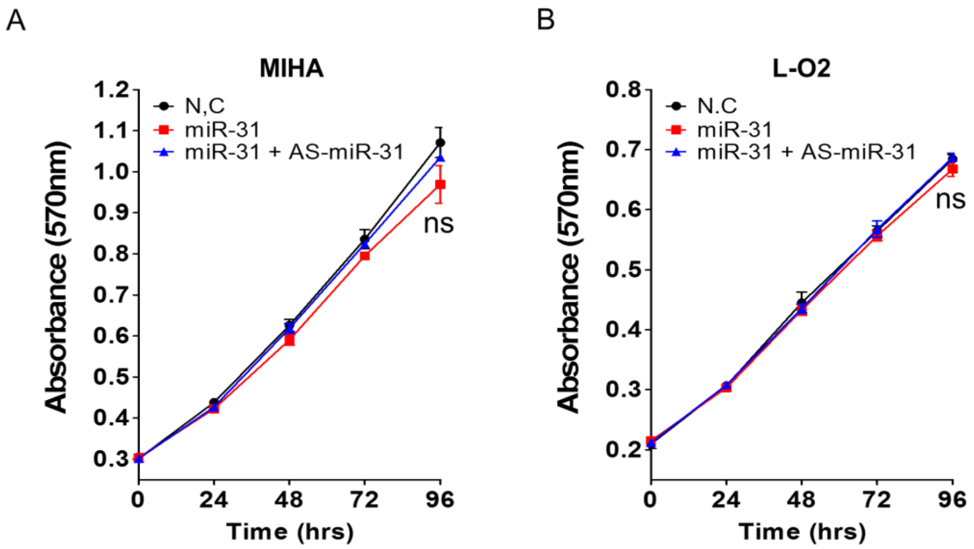

Supplementary Figure S2: Effect of ectopic expression of miR-31 in MIHA and L-O2 cells. (A and B) After transfection of miR-31 in MIHA and L-O2 cells, the cell viability was measured at A570. Cell growth was measured at every 24 hours. N.C represents negative control miRNA (ns; non significance).

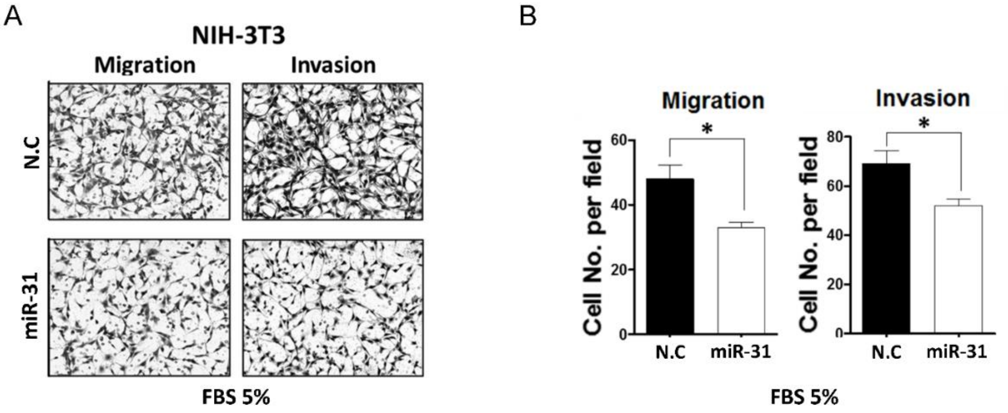

Supplementary Figure S3: Ectopic expression of miR-31 significantly suppressed migratory and invasive responses in ras-NIH-3T3 cells (A) Motility and invasion assays were performed after transfection using scrambled microRNA control and miR-31 to *ras*-NIH-3T3 cells as a pilot study. Representative images of the membranes of motility or invasion assay with invert microscope (magnification, x200). (B) The number of migrated or invaded cells were counted in randomly selected fields and presented in bar graph (means ± SD; *$P<0.05$, Student's *t* test).
